# Favorable mortality-to-incidence ratios of kidney Cancer are associated with advanced health care systems

**DOI:** 10.1186/s12885-018-4698-6

**Published:** 2018-08-06

**Authors:** Wen-Wei Sung, Shao-Chuan Wang, Tzuo-Yi Hsieh, Cheng-Ju Ho, Cheng-Yu Huang, Yu-Lin Kao, Wen-Jung Chen, Sung-Lang Chen

**Affiliations:** 1 Department of Urology, Chung Shan Medical University Hospital, No.110, Sec. 1, Jianguo N. Rd., South Dist, Taichung City, 402 Taiwan; 2 School of Medicine, Chung Shan Medical University, No.110, Sec. 1, Jianguo N. Rd., South Dist, Taichung City, 402 Taiwan; 3 Institute of Medicine, Chung Shan Medical University, No.110, Sec. 1, Jianguo N. Rd., South Dist, Taichung City, 402 Taiwan; 4 Department of Medical Education, Chung Shan Medical University Hospital, No.110, Sec. 1, Jianguo N. Rd., South Dist, Taichung City, 402 Taiwan; 5 Department of Urology, National Taiwan University Hospital, No.95, Wenchang Rd., Shilin Dist, Taipei City, 111 Taiwan

**Keywords:** Kidney cancer, Mortality, Incidence, Mortality-to-incidence ratio

## Abstract

**Background:**

The advancements in cancer therapy have improved the clinical outcomes of cancer patients in recent decades. However, advanced cancer therapy is expensive and requires good health care systems. For kidney cancer, no studies have yet established an association between clinical outcome and health care disparities.

**Methods:**

We used the mortality-to-incidence ratio (MIR) for kidney cancer as a marker of clinical outcome to compare World Health Organization (WHO) country rankings and total expenditures on health/gross domestic product (e/GDP) using linear regression analyses.

**Results:**

We included 57 countries based on data from the GLOBOCAN 2012 database. We found that more highly developed regions have higher crude and age-standardized rates of kidney cancer incidence and mortality, but a lower MIR, when compared to less developed regions. North America has the highest crude rates of incidence, but the lowest MIRs, whereas Africa has the highest MIRs. Furthermore, favorable MIRs are correlated with countries with good WHO rankings and high e/GDP expenditures (*p* < 0.001 and *p* = 0.013, respectively).

**Conclusions:**

Kidney cancer MIRs are positively associated with the ranking of health care systems and health care expenditures.

**Electronic supplementary material:**

The online version of this article (10.1186/s12885-018-4698-6) contains supplementary material, which is available to authorized users.

## Background

Cancer is a leading cause of death worldwide, and the burden continues to increase in both developed and less developed countries due to lifestyle behaviors, such as smoking, poor diet, and physical inactivity [1, 2]. Kidney cancer currently ranks as the seventh most common cancer in men and the tenth most common in women [3]. In 2012, the worldwide estimates for kidney cancer were 338,000 new cases (incidence: 2.4%) and 143,000 deaths (mortality: 1.7%) [3]. The geographic distribution of kidney cancer is highest in the Baltic countries and in Eastern European countries, such as the Czech Republic and Slovakia, and lowest in Africa and Asia, with the exception of Israel [4]. The mortality distribution also follows incidence patterns, with the highest death rates observed in Eastern Europe [4]. Renal cell carcinoma accounts for more than 90% of kidney malignancies, with the main subtype being clear cell renal cell carcinoma (70%) [5].

The clinical outcomes of cancer treatment can be measured by the five-year survival rate, as well as partially by the mortality-to-incidence ratio (MIR) [6–12]. In the past 10 years, the incidence of renal cell carcinoma has increased in most countries [13]. By contrast, the mortality associated with this disease has been relatively stable worldwide, but is decreasing in Western Europe, the US, and Australia [13]. For example, the five-year relative survival rate for kidney cancer patients in the US in 2005–2011 was approximately 74%, an increase from the rate of approximately 57% in the 1980s [1, 2]. These trends suggest that health care systems and health care expenditures are affecting the screening, treatment, and prognosis of kidney cancer.

We hypothesize that the MIR should be low in countries with better health care systems. Our primary goal in the present study was to identify the roles played by the level of human development, World Health Organization (WHO) rankings, and total expenditure on health/gross domestic product (e/GDP) in kidney cancer outcomes. Our secondary goal was to clarify the correlation between MIRs and the WHO ranking and e/GDP and to determine the association between e/GDP or WHO ranking and the crude rate or age-standardized rate (ASR) of kidney cancer incidence and mortality. Our results provide a general overview of the connection between MIR and health care disparities across countries.

## Methods

The data were acquired as described previously [6, 12, 14]. In brief, the cancer epidemiologic data were obtained from the GLOBOCAN 2012 database, which is maintained by the International Agency for Research on Cancer (https://www.iarc.fr/) [3]. Health care expenditures and life expectancies were obtained from the WHO World Health Statistics 2015, and the WHO rankings were obtained from the WHO World’s Health Systems. We included 184 countries listed in the GLOBOCAN 2012 database. Countries that lacked WHO ranking data (22 countries) or that had little data available (a ranking of E–G for incidence or a ranking of 4–6 for mortality; 105 countries) were excluded.

The MIR is defined as the ratio of the crude rate of mortality to the disease incidence [7, 10]. The method of statistical analyses was described previously [6, 14]. We used linear regression and SPSS statistical software (SPSS, version 15.0, Inc., Chicago, IL, US) to evaluate the association between the MIRs and variants. *P* values < 0.05 were considered statistically significant. Scatter plots were produced using Microsoft Excel 2010.

## Results

### The incidence and mortality of kidney cancer are higher in more developed regions and in regions in the west

We first sought to understand the present global situation regarding kidney cancer by analyzing the crude rate and the ASR of kidney cancer incidence and mortality according to development level, WHO region, and continent (see Table 1). The crude rate of incidence and the cancer-related mortality rate worldwide are 4.8 and 2.0, respectively, for kidney cancer. Both rates tend to be higher in more developed regions (incidence: 16.1 vs. 2.4; mortality: 6.0 vs. 1.2, respectively). The analysis based on WHO regions and continents indicated that the WHO European region had the highest crude rate of incidence and mortality (13.5 and 5.9, respectively), followed by the WHO Americas region (8.9 and 2.9, respectively). North America had the highest crude rate of incidence (18.2), and Europe had the highest mortality rate (6.6). The ASR distribution showed a similar pattern, as the ASRs of incidence and mortality were 9.2 and 2.8 in more developed regions, with the highest values associated with the WHO European region (8.3 and 3.1, respectively) and the WHO Americas region (7.3 and 2.2, respectively). North America had the highest ASR of incidence (11.7), while Europe had the highest ASR of mortality (3.1), and both regions are developed.

**Table 1:** Summary of the number of cases, rates, and mortality-to-incidence ratios of kidney cancer according to region

Region	Number		Crude rate		Age-standardized rate		Mortality-to-incidence ratio^a^
	Incidence	Mortality	Incidence	Mortality	Incidence	Mortality	
World	337,860	143,406	4.8	2.0	4.4	1.8	0.42
Development							
More developed regions	199,991	74,948	16.1	6.0	9.2	2.8	0.37
Less developed regions	137,869	68,458	2.4	1.2	2.6	1.3	0.50
WHO region categories							
WHO Africa region	6725	5649	0.8	0.6	1.0	0.8	0.75
WHO Americas region	85,005	27,949	8.9	2.9	7.3	2.2	0.33
WHO East Mediterranean region	8952	6628	1.4	1.1	1.9	1.5	0.79
WHO Europe region	121,629	52,816	13.5	5.9	8.3	3.1	0.44
WHO South-East Asia region	17,050	11,399	0.9	0.6	1.1	0.7	0.67
WHO Western Pacific region	98,473	38,951	5.3	2.1	4.1	1.5	0.40
Continent							
Africa	10,033	8169	0.9	0.8	1.2	1.0	0.89
Latin America and Caribbean	21,183	11,308	3.5	1.9	3.5	1.8	0.54
Northern America	63,822	16,641	18.2	4.7	11.7	2.6	0.26
Asia	123,402	57,058	2.9	1.3	2.8	1.3	0.45
Europe	115,252	49,025	15.5	6.6	8.8	3.1	0.43
Oceania	4168	1205	11.0	3.2	8.0	2.0	0.29

^a^the percentage in the ratio of the crude rate of mortalities and the crude rate of incidences

### The kidney cancer mortality-to-incidence ratios are high in less developed regions

We also investigated the MIRs to determine any association between this ratio and the outcomes of kidney cancer patients. The global kidney cancer MIR is 0.42, with a higher rate in less developed regions (0.5). The WHO East Mediterranean region had the highest kidney cancer MIR (0.79), followed by the WHO Africa region (0.75). Among the continents, Africa had the highest MIR (0.89). High MIRs were therefore associated with less developed regions and with Africa.

### World Health Organization ranking and total expenditure on health/GDP are significantly associated with kidney cancer mortality-to-incidence ratios

**Fig. 1:**
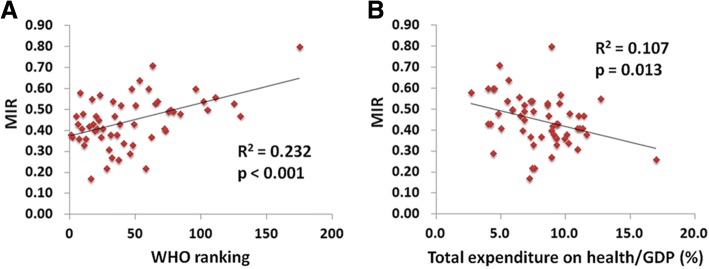
The (**a**) World Health Organization country rankings and (**b**) total expenditures on health/GDP are significantly associated with the mortality-to-incidence ratio of kidney cancer

We sought to understand the observed differences between nations by including countries based on national data, WHO rankings, total expenditure on health/GDP (e/GDP), crude rate of incidence and mortality, the ASR of incidence and mortality, and life expectancy (Table 2). France was the highest WHO ranked country, whereas the US had the highest e/GDP (17.0%). Among all the countries, the Czech Republic had the highest crude rate of incidence (22.7), and Estonia had the highest mortality rate (10.6). Of the 57 countries compared, Luxembourg had the lowest MIR (0.17). We further examined the correlation between the kidney cancer MIR and the WHO ranking and e/GDP (Table 2; Fig. 1). The WHO ranking and e/GDP showed a significant positive correlation with kidney cancer MIRs (*R*^*2*^ = 0.232, *p* < 0.001; *R*^*2*^ = 0.107, *p* = 0.013, respectively; Fig. 1).

**Table 2:** Summary of World Health Organization country rankings; total expenditure on health/GDP; life expectancy; and the kidney cancer incidence, mortality, and mortality-to-incidence ratios of selected countries

Country	Ranking	Total expenditure on health/GDP (%)	Life expectancy	Number		Crude rate		Age-standardized rate		Mortality-to-incidence ratio^a^
				Incidence	Mortality	Incidence	Mortality	Incidence	Mortality	
France	1	11.6	82	11,023	4186	17.4	6.6	9.7	2.8	0.38
Italy	2	9.2	83	11,300	4203	18.5	6.9	8.7	2.5	0.37
Malta	5	8.7	81	57	27	13.6	6.4	8.0	3.0	0.47
Singapore	6	4.2	83	401	175	7.6	3.3	5.2	2.2	0.43
Spain	7	9.3	83	6474	2295	13.8	4.9	7.9	2.2	0.36
Oman	8	2.7	76	36	21	1.2	0.7	2.1	1.4	0.58
Austria	9	11.1	81	1322	536	15.7	6.4	8.0	2.5	0.41
Japan	10	10.3	84	16,830	8124	13.3	6.4	5.3	1.9	0.48
Norway	11	9.3	82	798	263	16.1	5.3	9.3	2.5	0.33
Portugal	12	9.9	81	1004	368	9.4	3.4	5.0	1.4	0.36
Iceland	15	9.0	82	45	19	13.7	5.8	8.8	3.2	0.42
Luxembourg	16	7.2	82	70	12	13.4	2.3	8.3	0.9	0.17
Netherlands	17	12.7	81	2679	1463	16.0	8.8	8.8	4.0	0.55
United Kingdom	18	9.3	81	9714	4150	15.5	6.6	8.2	3.0	0.43
Ireland	19	8.9	81	571	230	12.5	5.0	8.4	3.0	0.40
Switzerland	20	11.4	83	948	448	12.3	5.8	6.5	2.4	0.47
Belgium	21	10.9	80	1763	728	16.3	6.7	8.7	2.7	0.41
Colombia	22	6.8	78	1048	483	2.2	1.0	2.4	1.1	0.45
Sweden	23	9.6	82	1125	635	11.8	6.7	6.4	2.6	0.57
Cyprus	24	7.3	82	46	17	4.1	1.5	3.0	1.0	0.37
Germany	25	11.3	81	18,615	7540	22.7	9.2	10.6	3.3	0.41
Israel	28	7.4	82	1002	217	13.0	2.8	10.0	1.8	0.22
Canada	30	10.9	82	5579	1739	16.1	5.0	9.3	2.5	0.31
Finland	31	9.1	81	882	333	16.3	6.2	7.9	2.4	0.38
Australia	32	8.9	83	3501	960	15.3	4.2	9.5	2.1	0.27
Chile	33	7.3	80	1353	737	7.8	4.2	6.0	3.1	0.54
Denmark	34	11.0	80	754	352	13.5	6.3	7.2	2.9	0.47
Costa Rica	36	10.1	79	179	69	3.7	1.4	3.7	1.4	0.38
United States of America	37	17.0	79	58,222	14,900	18.4	4.7	12.0	2.6	0.26
Slovenia	38	9.4	80	400	171	19.6	8.4	11.1	3.9	0.43
Cuba	39	8.6	78	517	271	4.6	2.4	3.1	1.5	0.52
New Zealand	41	10.2	82	586	198	13.1	4.4	8.2	2.4	0.34
Bahrain	46	4.4	77	23	7	1.7	0.5	2.6	1.0	0.29
Thailand	47	4.5	75	1017	632	1.5	0.9	1.2	0.7	0.60
Czech Republic	48	7.5	78	3313	1095	31.4	10.4	16.7	4.8	0.33
Malaysia	49	4.0	74	611	255	2.1	0.9	2.4	1.0	0.43
Poland	50	6.8	77	5244	2721	13.7	7.1	8.1	3.7	0.52
Jamaica	53	5.6	74	31	20	1.1	0.7	1.1	0.7	0.64
Korea, Republic of	58	7.6	82	5651	1264	11.6	2.6	8.0	1.6	0.22
Philippines	60	4.4	69	1008	600	1.0	0.6	1.4	0.9	0.60
Slovakia	62	8.1	76	1063	388	19.4	7.1	12.5	4.2	0.37
Egypt	63	4.9	71	1740	1275	2.1	1.5	2.4	1.8	0.71
Uruguay	65	8.6	77	465	243	13.7	7.2	9.4	4.4	0.53
Trinidad and Tobago	67	5.5	71	32	18	2.4	1.3	2.3	1.1	0.54
Belarus	72	5.0	72	1575	637	16.5	6.7	11.1	4.1	0.41
Lithuania	73	6.7	74	773	309	23.5	9.4	13.2	4.9	0.40
Argentina	75	6.8	76	4068	1998	9.9	4.9	8.0	3.6	0.49
Estonia	77	5.9	77	284	142	21.2	10.6	11.7	4.6	0.50
Ukraine	79	7.5	71	5240	2542	11.7	5.7	7.5	3.4	0.49
Mauritius	84	4.8	74	53	25	4.0	1.9	4.2	2.2	0.48
Fiji	96	4.0	70	4	3	0.5	0.3	0.4	0.4	0.60
Bulgaria	102	7.4	75	881	470	11.9	6.4	6.9	3.3	0.54
Latvia	105	5.9	74	449	225	20.1	10.1	10.9	4.7	0.50
Ecuador	111	6.4	76	403	216	2.7	1.5	2.9	1.5	0.56
Brazil	125	9.5	75	6255	3291	3.2	1.7	3.0	1.5	0.53
Russian Federation	130	6.5	69	19,313	9025	13.5	6.3	8.9	3.8	0.47
South African Republic	175	8.9	60	506	420	1.0	0.8	1.2	1.1	0.80

^a^the percentage in the ratio of the crude rate of mortalities and the crude rate of incidences

### No significant correlation is evident between the World Health Organization ranking, crude rate, and age-standardized rate of incidence and mortality for kidney cancer

Unexpectedly, we found no significant correlation between WHO ranking and the crude rate of incidence and mortality for kidney cancer (*R*^*2*^ = 0.058, *p* = 0.071; *R*^*2*^ = 0.018, *p* = 0.317, respectively; Additional file 1: Figure S1A and B). Countries with a higher WHO ranking also showed no higher incidence or greater mortality rate in age-standardized groups (*R*^*2*^ = 0.032, *p* = 0.185; *R*^*2*^ = 0.004, *p* = 0.629, respectively; Additional file 1: Figure S1C and D).

### The association between total expenditure on health/GDP and the kidney cancer crude rate and age-standardized rate of incidence and mortality

We also analyzed the correlation between e/GDP and crude rate and the ASR of incidence and mortality for kidney cancer (Additional file 2: Figure S2). The crude rate of incidence and mortality in these countries increased with increasing e/GDP (*R*^*2*^ = 0.237, *p* < 0.001; *R*^*2*^ = 0.169, *p* = 0.001, respectively; Additional file 2: Figure S2A and B), and the same trend was seen for the association between e/GDP and the ASR of incidence (*R*^*2*^ = 0.187, *p* = 0.001; Additional file 2: Figure S2C). However, no significant correlation was noted between e/GDP and the ASR of mortality (*R*^*2*^ = 0.053, *p* = 0.084; Additional file 2: Figure S2D). In summary, the e/GDP had a significant correlation with the incidence and mortality crude rate of kidney cancer, while the ASR of mortality was not significantly correlated with e/GDP.

## Discussion

To the best of our knowledge, this is the first article to explore the relationship between the MIRs of kidney cancer and WHO rankings, life expectancy, and e/GDP. Negative correlations between the WHO ranking and life expectancy and e/GDP (%) would be understandable, as disability-adjusted life expectancy and fair financial contribution were two of the index factors on which the WHO ranking is based. High MIRs are observed in less developed countries for genitourinary malignancies [15]. In the present study, we found a positive correlation between WHO rankings and MIRs, in agreement with a previous study on colorectal cancer that showed similar results among the Organisation for Economic Co-operation and Development countries [10]. Sunkara et al. attributed this correlation to the better screening programs provided by countries with better WHO rankings for certain cancer such as colorectal cancer. However, there is no screen program for kidney cancer, the improved MIR might relate to the availability of medical service and health examination.

As with colorectal cancer, kidney cancer outcomes depend on early detection and proper intervention. The increased demand for abdominal imaging has led to an increase in the incidental detection of kidney masses, usually as small indolent cancers [16]. As a result, in the US, 63% of kidney cancers are diagnosed at a localized stage [17], and this directly affects outcome as the five-year survival rates show substantial differences among stages. The localized stage has the best prognosis, with a 92% five-year survival rate, while the distant stage has only a 12% five-year survival rate [17]. These numbers point to the importance of early detection of this disease. In general, this means that countries with better health care programs would be expected to have lower MIRs due to the availability of image survey such as sonography or computed tomography scan. This increases the incidental finding of renal mass and might relate to early diagnosis and good prognosis. This could then explain the observed association between WHO rankings and MIRs.

We also found negative correlations between WHO rankings and crude mortality and incidence rates, indicating higher rates in countries with better WHO rankings. One possible explanation is the inconsistency of access to medical care among different countries, as nations with worse WHO rankings are less likely to have good health care access. This means less abdominal imaging and less detection of early signs and symptoms of kidney cancer, so the incidence and mortality rates increase. Another explanation is the age distribution of this disease. Most cases are diagnosed between the ages of 60 and 70, with the median age being 65 [18, 19]. Therefore, the populations of countries with longer life expectancy would have a greater risk of developing kidney cancer. As life expectancy correlates positively with WHO rankings and WHO rankings correlate negatively with mortality, the crude rates of incidence are understandable.

The impact of high health care expenditure on good MIRs for kidney cancer is multifactorial, as noted for other types of cancer [6, 11, 12, 14]. Patients in countries with higher health care expenditure would have a greater chance of early malignancy detection and prompt curative treatment or less invasive surgery. From the perspective of surgical intervention for kidney cancer, patients with early T stage cancer would have a larger volume of healthy renal parenchyma for renal preservation, which might result in a better clinical outcome [20]. For partial nephrectomy, outcomes are more favorable for robotic surgery than for laparoscopic surgery in terms of a lower conversion rate to radical nephrectomy, favorable retention of renal function, and shorter warm ischemia time [21–23]. These features could partially explain the role of health expenditure in the MIR of kidney cancer.

Our study has some limitations. Since the GLOBCAN database provides national statistics information worldwide, the data quality should be further validated. Countries with low data quality or unknown data quality were excluded to avoid misleading effects of over diagnosis or other influences. Due to concerns about generating misleading MIRs, we did not include all the countries listed in the database. This resulted in incomplete data, which makes our results unreliable in the global context. Furthermore, we did not document the diagnosed stage and risk factors among countries, such as smoking, obesity, and hypertension rates. These risk factors may play crucial roles in explaining the incidence and mortality rates among countries and regions. In addition, we only examined cross-sectional data for a single year, so the actual disease trend may not be accurately presented. Another limitation is the use of WHO rankings. This grading system was established in 2000, so it may not precisely reflect the current situation for health care systems in different countries, although the correlations with life expectancy and e/GDP speak to its credibility.

Despite these limitations, our study shows higher kidney cancer incidence and mortality rates in more developed regions and in countries with better WHO rankings. Moreover, the MIRs for these countries are negatively correlated with their WHO rankings for both genders. Based on the results, we suspect that the kidney cancer MIR might be an appropriate indicator for evaluating health care systems. The massive discrepancies in kidney cancer MIRs between countries and regions suggest a role for early detection and proper screening facilities in countries with higher MIR values.

## Conclusions

Kidney cancer MIRs are associated with the ranking of health care systems and health care expenditures and therefore might be an indicator of health care disparities.

## Additional files

Additional file 1: **Figure S1.** The association between the World Health Organization country ranking of total expenditures on health/GDP and the crude rates of (A) kidney cancer incidence and (B) kidney cancer-related mortality. The age-standardized rates of (C) kidney cancer incidence and (D) kidney cancer-related mortality. (TIF 282 kb)

Additional file 2: **Figure S2.** The association between the total expenditures on health/GDP and the crude rates of (A) kidney cancer incidence and (B) kidney cancer-related mortality. The age-standardized rates of (C) kidney cancer incidence and (D) kidney cancer-related mortality (TIF 298 kb)

## Abbreviations

ASR: Age-standardized rate
e/GDP: Total expenditures on health/gross domestic product
MIR: Mortality-to-incidence ratio
WHO: World Health Organization
