# Supplementary figures and images for: Favorable mortality-to-incidence ratios of kidney Cancer are associated with advanced health care systems
Source: BMC Cancer. 2018 Aug 6;18:792. doi: 10.1186/s12885-018-4698-6 (PMC6091202; doi:10.1186/s12885-018-4698-6)

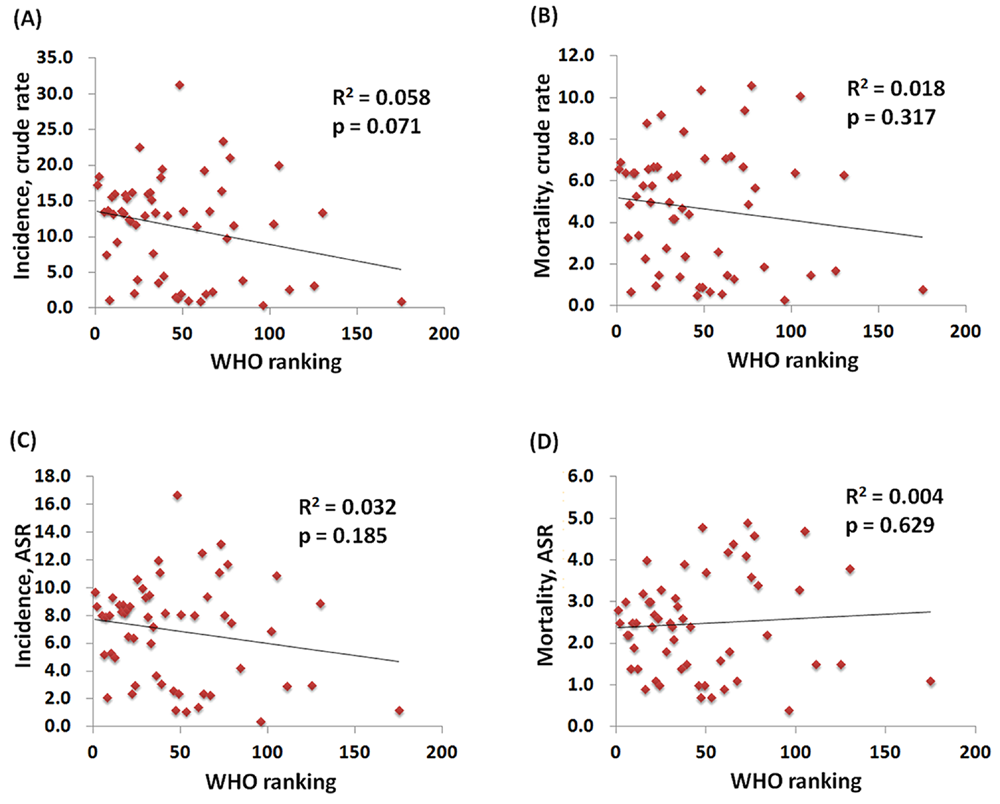

Supplement: Supplementary file 1 — Figure S1. The association between the World Health Organization country ranking of total expenditures on health/GDP and the crude rates of (A) kidney cancer incidence and (B) kidney cancer-related mortality. The age-standardized rates of (C) kidney cancer incidence and (D) kidney cancer-related mortality. (TIF 282 kb) [file 12885_2018_4698_MOESM1_ESM.tif]

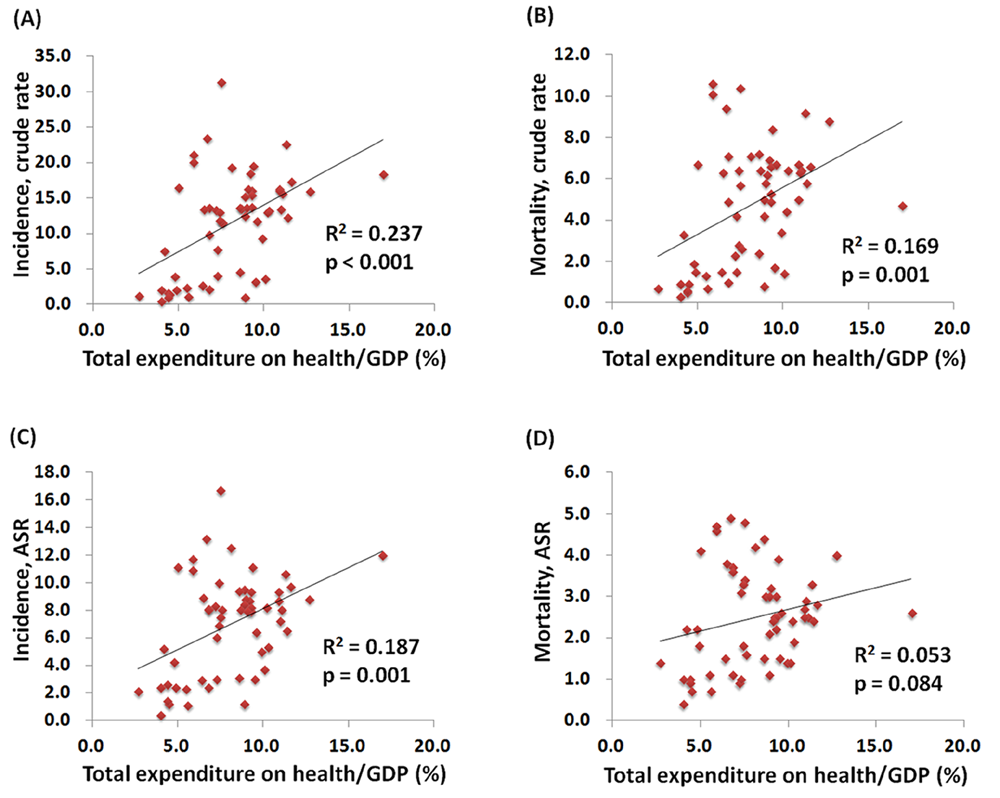

Supplement: Supplementary file 2 — Figure S2. The association between the total expenditures on health/GDP and the crude rates of (A) kidney cancer incidence and (B) kidney cancer-related mortality. The age-standardized rates of (C) kidney cancer incidence and (D) kidney cancer-related mortality (TIF 298 kb) [file 12885_2018_4698_MOESM2_ESM.tif]
